# Sexual Practice and Perception of HIV/AIDS Amongst Men who have Sex with Men in Kolkata

**DOI:** 10.4103/0970-0218.55285

**Published:** 2009-07

**Authors:** Soumya Deb, Sinjita Dutta, Aparajita Dasgupta, Biswajit Biswas

**Affiliations:** Department of Community Medicine, IPGMER, SSKM Hospital, Kolkata, India; 1 Department of Preventive and Social Medicine (PSM), All India Institute of Hygiene and Public Health, 110, C.R.Avenue, Kolkata - 700 073, India

**Keywords:** Anal sex, HIV, MSM, sex partner, sexual practice

## Abstract

**Background and Objectives:**

Men who have Sex with Men (MSM) are a vulnerable population and need special attention in the fight against the global pandemic of HIV/AIDS. A study was conducted in an MSM clinic to find out to their varied socio-demographic characteristics, their knowledge and attitude towards HIV/AIDS, and its association with their different sexual practices.

**Materials and Methods:**

Descriptive, cross sectional study conducted in an MSM clinic in central Kolkata.

**Results:**

A total of 108 MSM were studied over a period of six months. A majority (25%) were students, followed by drivers (22.2%), with mean age being 22.8 years. About 13.9% of them were illiterate and 30.6% of them married. A majority (75%) of the clients were initiated to first sexual act during adolescence. Most (44%) of them had indulged in sexual acts with two/three partners in the past one month. The most common form of sexual act was receptive anal sex (83.3%). The commonest reasons for indulging into such sexual acts with men were increased pleasure to have sex with men and increased sexual urge (38.9% and 27.8%) while 19.4% performed such acts in an intoxicated state. Only 22.2% ever used condom in the last one month during sexual acts. Their knowledge and positive attitude towards HIV/AIDS increased significantly with increase in literacy status (*P* less than 0.01). Only 36 (33.3%) knew about HIV transmission through anal route while only 35.2% knew the correct method to use condom. Favorable sexual practices like using a condom or having fewer partners was more among the literates than the illiterates (*P* less than 0.05). Alarmingly 44.4% felt that one should have sex without a condom if his sex partner was extremely attractive, 88.9% felt that using a condom was not necessary if his partner was clean and hygienic, 69.4% felt that anal sex is for fun, so no condom is required while 43.5% felt getting HIV was a matter of bad luck.

**Conclusion:**

Proper IEC to promote condom use and promotion of safe sexual practice among MSM is the need of the hour.

## Introduction

Next to South Africa and Nigeria, India has the third largest number of persons living with HIV/AIDS in the world.(1) According to the data on the National AIDS Control Organization, adult HIV prevalence in India is approximately 0.36 percent, amounting to between two and 3.1 million people.(2) It is also estimated that 85% of HIV transmission is sexual. Though the prevalence of HIV/AIDS among MSM in India was officially set at 5.69% percent in 2006,(2) it is felt by the researchers that this figure underestimates the impact of unsafe sexual practice of the MSM on the epidemic of HIV/AIDS in India, especially since global estimates suggest that five per cent to 10 per cent of HIV prevalence is attributable to sexual transmission between men.(1) Truck drivers are a group known to have higher levels of homosexual behavior than the general public.(3) Therefore, the high rates of HIV infection among truck drivers may be an indicator of the importance of homosexual transmission in the India epidemic because homosexual behavior also takes place outside of this particular group.(4)

The reliability of HIV infection data among Men Who Have sex with Men (MSM) is influenced by: (i) the lack of knowledge and understanding of MSM behavioral patterns as many MSM do not have a conscious sexual identity/orientation; the fact that (ii) many do not consider reporting on their same sex behaviors even when asked; (iii) many do not identify their sexual behavior as MSM since their partners are not perceived as men; (iv) many gay-identified men as well as others who have developed a sexual identity, are reluctant to identify themselves and disclose their same-sex behaviors or sexual orientation to health care providers, fearing stigma, discrimination and exclusion.

The framework of male to male sex is substantially divergent and inclusive. ‘Men who have sex with men (MSM)’ denotes men who have sex with other men regardless of the presence or absence of any specific sexual identity.(5) It includes normative males who desire to penetrate as the only signifier, feminized males (*kothis*) who desire to be penetrated by other males, *hijras* who are born as biological or anatomical males but reject their masculine identity in due course of time to identify, either as women or not-men, or in between man and woman, or neither man or woman (almost like *Kothis*) and adolescent and other males who desire to experiment for fun.(5) Males are often easier to access for sex than females, while male sex workers are usually cheaper than female sex workers are. MSM includes self-identified gay men, primarily among the urban, English speaking elite and middle classes. Without a welfare system, and with significant levels of unemployment or low level incomes, male sex work can be a way out in terms of supporting the self and family. This is not to imply that males involved in sex work do not enjoy the sex with other males. Often they may also have a regular male partner, and/or a wife or girlfriend. Thus, the MSM is a very high risk group and acts as a bridge in transmission of sexually transmitted disease to the general population.

There is definitely an insensitive attitude towards this population leading to their social exclusion and deprivation of service provision, treatment and care. Again, an underestimate of the number of at-risk MSM in any given population apparently leads to lack of resources to support HIV intervention programs exclusively for this vulnerable population. No doubt these people live in a world of their own with misconceptions and wrong notions regarding sexually transmitted diseases especially HIV/AIDS with little access to health education regarding these issues.

With this in the backdrop, the present study was conducted in an MSM clinic in Central Kolkata. The clinic is run under the auspices of an NGO, Manas Bangla, which provides care and treatment to the MSM in eastern India especially West Bengal.

## Materials and Methods

A descriptive, cross sectional study was conducted among MSM attending a MSM clinic in Central Kolkata. The clinic is located in Kadapara area of Central Kolkata and is frequented by MSM and other male clients as well. The clinic runs on Mondays, Wednesdays and Fridays of every week. The objectives of the study were as follows:

1. To find out the different socio-demographic characteristics of the study population
2. To elicit the their perception of HIV/AIDS
3. To find out their pattern of sexual practice
4. To find out the association, if any, of the knowledge and attitude of the study population with their literacy status and sexual practices

The different variables under study included socio-demographic characteristics like age, literacy status, occupation, length of stay, living arrangement, marital status and per capita monthly income (PCI); different aspects of knowledge regarding HIV/AIDS like ever heard about the disease, its varied modes of transmission (checklist given), seriousness of problem, curability of the disease, heard of condom and knowing correct use of condom. Scoring regarding their knowledge was decided as follows - *yes +1, no −1, except for the last question i.e. knowing the method of using condom where Correct knowledge was assigned+1, incorrect −1*, Do Not Know (DNK) 0, (thus maximum possible score was plus15 and minimum score attainable was minus 12).

Besides, the following enquiries were made to find out the attitude of the study population-

1. If the man is sterilized, there is no need to wear condom
2. Condoms are only for family planning purposes
3. It is not necessary to use condom with male sex partner
4. I may decide to have sex without a condom if my sex partner is extremely attractive
5. If my partner is extremely clean and hygienic, then using a condom is not necessary
6. One cannot have proper sex while using a condom
7. Anal sex is just for fun, so no condom is required
8. If both partners have HIV, there is no need to use condoms
9. Condoms can cause premature ejaculation
10. Whether or not we use a condom during anal sex is up to my male partner
11. My partner and I will use a condom if I tell him we should
12. My partner encourages me to use condoms
13. I encourage my partner to use condoms
14. HIV only affects poor people
15. Getting HIV is a matter of bad luck
16. If a person has HIV/AIDS, his family relationships will be destroyed
17. If a person has HIV/AIDS, his relationship with friends will be destroyed
18. If a person has HIV/AIDS, his respect in the society will be destroyed

Correct attitude (+1), Incorrect attitude (−1), DNK 0, (Max score +18, Minimum Score − 18)

The varied sexual practices of the study population was also elicited by enquiring about their first sexual partner, age of first sex, ever used condom, use of condom during last sexual act, types of sexual acts practiced in last 12 months, types of sexual partners in the last 12 months, number of partners over the last one month and reasons for choosing a male partner for sexual act.

All MSM clients visiting the STD clinic during the study period of six months during 2007 were interviewed using a pre-designed semistructured questionnaire. The questions were taken from a research questionnaire approved by State AIDS Control Society of West Bengal. All the clients were interviewed after taking informed oral consent and on the basis of anonymity. A total of 108 MSMs visited the clinic with varied symptoms of sexually transmitted diseases like urethral discharge, perianal and penile warts, dysuria, ulcer on the glans, anal discharge as well as general complaints like fever, cough and cold and generalized body ache during the study period. These symptoms were treated as per syndromic approach in the clinic and referrals were made to the nearest medical college and hospital wherever felt necessary. All the attendees who visited the clinic during the study period willingly consented to the study. The results were analyzed at the end of the study using suitable statistical tests taking help of Microsoft Office Excel 2003 and EpiInfo version 3.2 softwares.

## Results and Discussion

Table 1 shows the socio-demographic characteristics of the study population. The mean age of the study population was only 22.1 years, most of them being in the age group 19 to 23 years. The literacy status was varied with 13.9% being illiterate while 16.7% were educated up to higher secondary and above. Many of the MSM (22.2%) had migrated from neighboring states of Jharkhand, Bihar, Orissa and UP and were staying in the locality for less than two years. The study population belonged to various occupations -unemployed, drivers, businessmen or professionals. However, what was extremely alarming was a majority (25%) of clients were students. Some students indulged in such practices while residing in mess and hostels. Almost one-third of the MSM were married. Besides, the per capita monthly income (PCI) was also found to be quite varied with 8.3% having a PCI of more than Rs 2500 while 25% had a monthly PCI of less than Rs 500.

**Table 1:** Socio-demographic characteristics of the study population (n=108)

Age	No. (%)	Mean + SD
14-18	27 (25)	16.0 ± 1.5
19-23	36 (33.3)	19.9 ± 0.9
24-28	21 (19.4)	25.1 ± 1.6
29-33	18 (16.7)	30.8 ± 1.2
34-38	3 (2.8)	36.0 ± 1.4
39-43	3 (2.8)	39.3 ± 0.4
Total	108 (100)	22.8 ± 6.3
**Literacy status**	**No.**	**%**
Illiterate	15	13.9
Just literate/below primary	21	19.4
Primary school completed	9	8.3
Middle school completed	18	16.7
Secondary completed	27	25
Higher secondary completed	11	10.2
Graduate	4	3.7
Post graduate and above	3	2.8
**Length of stay**	**No.**	**%**
<2 years	24	22.2
>2 years but not since birth	27	25
Since birth	57	52.8
**Occupation**	**No.**	**%**
Unemployed	5	4.6
Unskilled workers	11	10.2
Drivers	24	22.2
Shop owner	12	11.1
Petty traders	9	8.3
Self employed/professionals	17	15.7
Clerical/salesman	3	2.8
Student	27	25
**Living arrangement**	**No.**	**%**
Living alone	6	5.6
Living with family	75	69.4
Living with friends	27	25
**Marital status**	**No.**	**%**
Unmarried	75	69.4
Married	33	30.6
**PCI monthly income (in Rs)**	**No.**	**%**
<500	28	25.9
501-1000	30	27.8
1001-1500	18	16.7
1501-2000	10	9.3
2001-2500	13	12.0
>2500	9	8.3

Table 2 depicts the pattern of sexual practice of the study population. The age of first sexual act was found to be as low as 10 years in two instances. It was also observed that majority (44.2 %) had their first sexual act in the age group 15-19 years and the mean age of first such act only 16.6 years. As expected, the first sexual partner was a male friend in majority of the cases (50.9%) but it was also observed that CSW, girlfriends, classmates, relatives and even spouse at times was the partner in their first sexual act. The study population mostly had multiple partners with 58.2% having more than one partner in the last one month. Even in 5.6% of the cases, the number of partners in the last one month was alarmingly high in the order of six or more. Another interesting finding was the presence of male sex workers as sexual partners in 16 instances in the last one year or so. The most common sexual act was receptive anal sex followed by vaginal sex, oral sex and insertive anal sex. The use of condom was very poor with only 15.7% using the barrier method during vaginal intercourse and 6.5% during anal intercourse in the last one month. Among the study participants, only 22% had ever attended VCCTC and undergone the HIV test, only one of them was HIV positive. The HIV positive person was incidentally suffering from perianal carcinoma. It was found that majority i.e. 80 (74%) of the MSM who visited the clinic were feminized males practicing receptive anal sex (traditionally called *Kothis*) followed by *Pariks* (15, 14%) who considered themselves real males and practiced insertive anal sex, *Duplis* (8, 7%) who practiced both insertive and receptive form of sex and Eunuchs (five, five per cent) or the transsexuals traditionally called *Hijras*.

**Table 2:** Sexual practices practiced by the study population (n=108)

Age (in yrs) of first sexual act	No. (%)	Mean ± SD
10-14	33 (30.6)	12.2 ± 1.7
15-19	48 (44.4)	16.9 ± 1.3
20-24	24 (22.2)	21.1 ± 1.5
25-29	3 (2.8)	28.7 ± 0.9
Total	108 (100)	16.6 ± 4.0
**First sex partner**	**No.**	**%**
Male friend	55	50.9
Commercial female sex worker	18	16.7
Classmate	15	13.9
Girlfriend	9	8.3
Wife	6	5.6
Relative	5	4.6
**Number of sex partners in the last 1 month**	**No.**	**%**
0-1 partner	45	41.7
2-3 partners	48	44.4
4-5 partners	9	8.4
6-7 partners	3	2.8
8-9 partners	3	2.8
Sex partners over last 12 months*	**No.**	**%**
Male friend	107	99.1
Wife	33	30.6
Commercial female sex worker	14	13.0
Girlfriend	7	6.5
Hijra	17	15.7
Male sex workers	16	14.8
Nature of sexual intercourse*	**No.**	**%**
Receptive anal sex	90	83.3
Vaginal sex	54	50.0
Oral sex	48	44.4
Insertive anal sex	32	29.6
**Use of condom and VCCTC**	**No.**	**%**
Ever used condom in last one month during vaginal intercourse	17	15.7
Ever used condom in last one month during anal intercourse	7	6.5
Ever done HIV test	24	22.2
HIV status positive	1	0.9

* Multiple responses

Table 3 shows the association of knowledge score of the study population with factors like literacy status, condom use and number of partners in the last one month. Overall knowledge score of the study population was poor with 47.2% scoring less than three out of a maximum possible score of 15. Knowledge score significantly increased with the literacy status of the study population (*P* less than 0.05). The practice of ever using condoms in the last one month was significantly more (*P* less than 0.05) in men with higher knowledge score. There was however inverse relation between knowledge regarding HIV/AIDS and number of sex partners (*P* less than 0.01). Although all the MSM attending the clinic had heard about HIV/AIDS, only 36 of them (33.3%) knew about transmission through anal route while majority i.e. 61 (56.5%) knew about transmission through vaginal intercourse. Only 38 MSM (35.2%) knew the correct use of condom.

**Table 3:** Knowledge score of study population and association with other factors (n=108)

Knowledge score of the study population		
Knowledge score	No. (%)	Mean ± SD
0-3	51 (47.2)	1.7 ± 0.7
4-7	27 (25.0)	5.2 ± 1.0
8-11	30 (27.8)	9.7 ± 1.1
Total	108 (100)	4.8 ± 3.5
Association of knowledge score with literacy status		
Literacy status	Knowledge score <4 (%)	Knowledge score≥4 (%)
Illiterate (n=15)	12 (80)	3 (20)
Just literate/below primary (n=21)	15 (71.4)	6 (28.6)
Primary and Middle school completed (n=27)	15 (55.6)	12 (44.4)
Secondary and above (n=45)	9 (25)	36 (75)
Chi square value = 6.06, df=1, *P*<0.05, OR=4.86 (1.13 <OR <23.36)		
Association of knowledge score with condom use in the last one month		
Ever used condoms in last one month	Knowledge score <4 (%)	Knowledge score≥4 (%)
Yes (n=24)	6 (25)	18 (75)
No (n=84)	45 (53.6)	39 (46.4)
Chi square value = 6.11, df=1, *P*<0.05, OR=5.54 (1.32<OR <26.68)		
Association of knowledge score with number of partners in the last one month		
Number of partners	Knowledge score <4 (%)	Knowledge score≥4 (%)
0-1 partner (n=45)	12 (26.7)	33 (73.3)
2-3 partners (n=48)	30 (62.5)	18 (27.5)
4-5 partners (n=9)	6 (66.7)	3 (23.3)
>6 partners (n=6)	3 (50)	3 (50)
Chi square value = 13.08, df=1, *P*<0.01, OR=0.22 (0.09<OR <0.56)		

Table 4 depicts the attitude score of the study population and its association with the same factors (namely literacy status, condom use and number of partners in the last one month). Like knowledge, attitude score significantly increased with literacy status of the study population (*P* less than 0.05). The favorable practice of ever using condom in the last one month was significantly more among men with higher attitude scores. Some alarming findings included 48 (44.4%) felt that they will perform sexual act without a condom if their sexual partner is extremely attractive, 96 (88.9%) felt that using a condom is not necessary if the partner is clean and hygienic, 75 (69.4%) felt that anal sex is for fun, so no condom is required, 47 (43.5%) felt getting HIV was a matter of bad luck, 57 (52.8%) felt their respect in the society would be destroyed if they had HIV/AIDS while only 35 (32.4%) encouraged their partners to use condoms.

**Table 4:** Attitude score of study population and association with other factors (n=108)

Attitude score of the study population		
Attitude score	No. (%)	Mean ± SD
<−0.5 [median score]	54 (50)	−7.6 ± 3.6
>−0.5	54 (50)	3.8 ± 2.5
Total	108 (100)	−1.9 ± 6.5
Association of attitude score with literacy status		
Literacy status	Attitude score<−0.5	Attitude score≥−0.5
Illiterate (n=15)	12 (80)	3 (20)
Just literate/below primary (n=21)	15 (71.4)	6 (28.6)
Primary and Middle school completed (n=27)	15 (55.6)	12 (44.4)
Secondary and above (n=45)	12 (26.7)	33 (73.3)
Chi square value = 4.95, df =1, *P*<0.05, OR=4.86 (1.13 <OR <23.36)		
Association of attitude score with condom use in the last one month		
Ever used condoms in last one month	Attitude score <−0.5 (%)	Attitude score≥−0.5 (%)
Yes (n=24)	3 (12.5)	21 (87.5)
No (n=84)	51 (60.7)	33 (39.3)
Chi square value = 15.48, df=1, *P*<0.01, OR=0.09 (0.02 <OR <0.36)		
Association of attitude score with number of partners in the last one month		
Number of partners	Attitude score <−0.5 (%)	Attitude score≥−0.5 (%)
0-1 partner (n=45)	18 (40)	27 (60)
2-3 partners (n=48)	27 (56.3)	21 (43.7)
4-5 partners (n=9)	6 (66.7)	3 (23.3)
>6 partners (n=6)	3 (50)	3 (50)
Chi square value = 3.09, df=1, *P*>0.05, OR=0.50 (0.21 <OR <1.17)		

Table 5 shows the varied responses given by the study population when asked about the reason of such sexual acts performed by MSM. Interestingly majority (40%) of the study population had sex with men for pleasure, 28% felt it was because of increased sexual urge while 22% thought they couldn't resist their sexual urge. Another striking finding was that, 8.3% were forced into the act while 1.9% performed such acts for exchange of money. About 19% of the men did the act in an intoxicated state.

**Table 5:** Reasons of sexual acts with men performed by MSM (n=108)

Reasons*	Number (%)
Increased pleasure to have sex with men	42 (38.9)
Increased sexual urge	30 (27.8)
Could not resist temptation	24 (22.2)
Indulged in sexual acts with men in an intoxicated state	21 (19.4)
Partner kept insisting	18 (16.7)
No chance to have sex with a female partner	18 (16.7)
Forced to indulge into sexual act by male partner	9 (8.3)
Ditched by girlfriend	5 (4.6)
Got money	2 (1.9)

* Multiple responses

## Conclusion and Recommendations

The MSM attending the clinic came from diverse walks of life ranging from unemployed unskilled workers to professionals and students. The knowledge and attitude of these men regarding HIV/AIDS were in a deplorable state. Most of them had multiple sex partners and they never used condoms in the last one month. In another study conducted among MSM in four cities - Bangalore, Hyderabad, Puducherry and Sylhet, in 2000, nearly 78% to 96% of MSM were found to have multiple partners in the last one month. In the same study condom use was slightly higher (29% to 45%) than the current study (22.2%).(6) What was even more disturbing was that many of these men were married and served as the bridge population in the transmission of different sexually transmitted diseases including the HIV epidemic. It was quiet distressing to find that 25% of these MSM were students-who were young people who had a productive life and career to fulfill. Getting exposed to HIV/AIDS thus meant a great loss of the most productive population of the country. So from the above study, we conclude, that time has come to address the needs of this highly vulnerable group. The researchers cleared the misconceptions and counseled each member of the study population after the interview was completed. They were also taught the correct way of using condom taking help of clay models at the end of the study. However, extensive, effective and energizing IEC with the involvement of motivated peer group to improve the knowledge, attitude and sexual practices of this high risk group is the need of the hour.
